# Trajectories of Life Satisfaction and their Predictors among Korean Older Adults

**DOI:** 10.1186/s12877-017-0485-5

**Published:** 2017-04-19

**Authors:** Hyun Ja Lim, Dae Kee Min, Lilian Thorpe, Chel Hee Lee

**Affiliations:** 1 Department of Community Health and Epidemiology, College of Medicine, University of Saskatchewan, 107 Wiggins Road, Saskatoon, SK S7N 5E5 Canada; 2 Department of Information Statistics, Duksung Women’s University, Seoul, South Korea; 3 Clinical Research Unit, College of Medicine, University of Saskatchewan, Saskatoon, Canada

**Keywords:** Longitudinal study, Well-being, Korean Retirement and Income Study (KReIS), Latent class growth model

## Abstract

**Background:**

Among older adults, life satisfaction (LS) correlates with health, mortality, and successful ageing. As various potential threats to LS tend to increase with advancing years, patterns of age-related changes in LS among older adults remain inconsistent. This study aimed to identify LS trajectories in older adults and the characteristics of individuals who experience them.

**Methods:**

Large-scale, nationally representative, longitudinal data collected from 2005 to 2013 were analyzed for this study. The outcome measure was a summary of multidimensional domains influencing LS: health, finance, housing, neighbor relationships, and family relationships. Latent class growth models and logistic regression models were used to identify trajectory groups and their predictors, respectively.

**Results:**

Within 3517 individuals aged 65 or older, five trajectories were identified across eight follow-up years: “low-stable” (TG1; *n* = 282; 8%), “middle-stable” (TG2; *n* = 1146; 32.6%), “improving” (TG3; *n* = 75; 2.1%), “upper middle-stable” (TG4; *n* = 1653; 47%), and “high” (TG5; *n* = 361; 10.3%). High trajectory individuals more frequently had higher education, financial security, good physical health, and good mental health than those in the stable, but less satisfied, groups. Similarly, compared to the largest group (upper middle-stable trajectory), individuals in the low-stable or middle-stable trajectory group not only had poorer physical and mental health but were more likely to be living alone, financially stressed, and residing in urban locations. Individuals with improving trajectory were younger and in poorer mental health at baseline compared to the upper middle-stable trajectory group.

**Conclusion:**

Life satisfaction in the older follows distinct trajectories. For older adults, trajectories are stable over time and predictable, in part, from individual characteristics. Knowledge of these patterns is important for effective policy and program development.

## Background

Life satisfaction (LS) indicates a subjective well-being based on an individual’s happiness and quality of life. The Organization for Economic Cooperation and Development (OECD) states that it “measures how people evaluate their life as a whole rather than their current feelings” [1]. LS has been reported to vary over a life span with an inverted U-shape; it remains lower but stable before age 60, notably increasing after this time point, and subsequently decreasing late in life due to increased dependency, health problems, and loss of close relationships [2–6]. More specifically, LS has been reported to decline sharply among the adults over 70 years of age [7], more rapidly so as individuals approach death [8, 9]. Since LS is strongly associated with health and mortality among the older population, it has been directly related to the concept of successful ageing and thus is viewed as a comprehensive indicator of successful aging [10].

Published literature suggests that diverse factors are associated with LS, including age, sex, education, marital status, religion, household environment, residential location, economic status, physical and mental health, and family and social supports [10–20]. Older adults who have retained their physical abilities and can perform activities of daily living tend to have higher LS, while those with mental health problems report lower life satisfaction [11, 21, 22]. Symptoms such as low mood, anxiety, and other indicators of psychiatric morbidity have been negatively correlated with LS, especially among older adults who live alone or have weak social support networks [10–12, 23, 24]. Literature also suggests that older individuals with serious medical illnesses, injuries, disability, physical inactivity, isolation, and recent relocation are more vulnerable to developing depression [23, 25].

Factors affecting social involvement and close personal relationships are also thought to have considerable influence on LS. For example, studies have shown that partnership is a significant predictor associated with well-being in older people [26, 27]. Older people (both males and females) perceive having a spouse as important to their overall life satisfaction trajectories. However, males have been found to be somewhat less satisfied with life when living without their spouse compared to females [26]. In general, living alone results in lower LS than living with a spouse or family members. Older individuals residing with families have been found to have much higher life satisfaction compared to the institutionalized elderly [6]. Deterioration in health among older adults resulting in need for placement in long-term care and inadequate family support, is also related to decreasing life satisfaction. As both social supports and health status influence, and are influenced by, financial circumstances, it is not surprising that many studies also suggest that financial security is an additional important component of LS in the older population [14, 16, 28, 29].

As various potential threats to LS, such as deterioration in health and changes in social and living environment, tend to increase with advancing years, it is easily assumed that LS declines in older age. However, patterns of age-related changes in LS among older adults remain inconsistent. Some studies have found that increased age is positively correlated with LS [5, 30, 31], while others have reported a significant decline in LS with age [7–9]. Still other studies have found stable levels of LS with age [32, 33]. Reasons for these discrepancies may be related to both underlying characteristics of the elderly as well as methodological issues in the study of LS. For example, Hsieh [35] noted that the older population is more heterogeneous than any other age group, potentially leading to inconsistent associations with LS [34], and most studies to date have been cross-sectional designs, which do not allow detection of the heterogeneity in LS over time. Although some studies have been longitudinal, cluster analysis and/or averaged group data have been typically used to examine LS changes over time, seeking to determine distinctive, pre-specified subgroup variations in the pattern of LS. To more fully identify the heterogeneity in groups, appropriate study designs and analytic methodology are required.

To the best of our knowledge, limited research has questioned how heterogeneity affects the age related patterns of change of LS in the elderly. Our primary hypothesis proposed that there are distinct trajectories of LS in older adults - subpopulations of elderly whose LS respectively decline, temporarily increase, or stabilize and improve. We also expected to find differing predictors for the distinct trajectories. Thus, our second hypothesis was that demographic, physical, mental, and environmental characteristics are predictors of different life satisfaction trajectories.

The aims of our study were to identify differential trajectories of LS in older adults, to determine subpopulations for which LS varies, and to identify predictors for distinguishing between trajectories. The planned analysis was to first determine whether different groups of participants showed different satisfaction trajectories and how many different trajectory subgroups exist. We then intended to identify predictors of membership in the different trajectory groups.

## Methods

### Data and Sample

This longitudinal study describes the eight year trajectories of life satisfaction among the older population in Korea. The data for this study was obtained from the Korean Retirement and Income Study (KReIS), a longitudinal survey conducted biennially from 2005 to 2013.

The KReIS aimed to investigate employment status, retirement, health, family relationships, and household economic situation of the elderly, providing an ongoing assessment of factors that potentially influence the degree of preparation for, and satisfaction during, older life. In the KReIS, the nationwide target population was general households with at least one member over 50 years of age. The initial sample was extracted from the sub-population of 24,995 general survey sites (1,420,299 households) among 10% of the Korean Population and Housing Census in 2000. Briefly, the KReIS used a stratified sampling frame and a total of 8567 individuals aged 50 or older participated in the initial survey in 2005. The core questions in the survey covered a wide-range of topics, including demographic aspects, economic status, housing, retirement, health status, and satisfaction with life. Items related to LS were included in a subsection in the survey. Additional descriptions of the study design and profile have been published elsewhere [35]. For our study, baseline responses from individuals aged 65 or older at the initial survey were examined, as were their subsequent responses for each wave that followed if answers to satisfaction items were provided. A total of 3517 individuals in the initial 2005 survey met our study criteria. Demographic and other data were collected at each time point over eight years (at 2005, 2007, 2009, 2011, and 2013).

### Measures

Various instruments have been used to measure LS, and these have commonalities as well as differences (see, for example, the article by Diener [36]). Differences in culture, age, and even gender might affect the appropriateness of specific selections. The measure of LS we used in this study was based on review of the literature pertaining to LS, but was also shaped by the available data in the KReIS data set.

We considered LS to be the sum of satisfaction scores within five domains: health, finance, housing, neighbor relationships, and family relationships. The question of each LS domain was scored on a 5-point Likert scale that asked, “To what extent are you satisfied with the item below?”, evaluated on a scale ranging from 1 to 5 (very unsatisfactory =1, unsatisfactory = 2, fair = 3, satisfactory = 4, very satisfactory =5). An individual’s LS outcome was calculated by summing the scores from those 5 domains, with a maximum score of 25. Higher scores indicated better life satisfaction.

Baseline covariates were measured in 2005; these included sex, age, education, marital status, residential area, number of members in the household, household composition type, housing type, current physical and mental health status, private health insurance, household income, and household expense. Age was categorized as 65–69, 70–74, 75–79, and 80 years and older. Sex was coded 0 = male and 1 = female. Education was coded as 0 = no education, 1 = Grade 1–6, and 2 = Grade 7 or higher. Residential area was categorized into two areas by population size: urban (population ≥ 50,000) was coded as 0 and rural (population < 50,000) was coded as 1. Household composition type was categorized as 1 = living alone, 2 = living with a spouse, and 3 = other mixed arrangements. Housing type was categorized as 1 = detached house, 2 = apartment, and 3 = other types. Current physical and mental health status were categorized as 1 = poor or very poor, 2 = fair, and 3 = good or very good. Participants’ economic status, household income, and expenses were available in the Korean currency Won ($1 USD ≈ 1100 Won). To standardize economic status, the household financial adequacy index (%), approximating the level of financial adequacy in a household [37], was calculated as.

$$ \frac{\left(\mathrm{household}\ \mathrm{income}\hbox{-} \mathrm{household}\ \mathrm{expense}\right)}{\mathrm{household}\ \mathrm{income}}\times 100. $$

A positive value indicates reasonable financial resources, while a negative value suggests financial difficulty.

### Statistical Analysis

Several analytical procedures were applied to the data. Descriptive statistics were used to summarize the characteristics of the study participants in the trajectory groups and are presented as means and standard deviations for continuous variables and percentages for categorized variables. ANOVA and the Chi-square test were used for trajectory group comparison of continuous and categorical variables, respectively.

For further analysis, we applied latent class growth modeling and logistic regression to the data.

In similar data analysis, other authors have used cluster analysis for determining differential subgroups [20]. However, cluster analysis does not allow us to obtain a detailed perspective on individual differences within subgroups. In contrast, latent class growth modeling (LCGM), based on regression and structural equation model, is more flexible than cluster analysis for identifying trajectories and heterogeneous subgroup population [38, 39]. Rather than evaluating individual time points or change between adjacent time points, LCGM identifies groups of subjects who have a similar outcome pattern over the study period as whole; thus, in our study LCGM was used to determine different trajectories of LS within the data. LCGM is a semi-parametric statistical method that was used to capture heterogeneous subpopulations in regard to the course of life satisfaction and to identify a number of meaningful classes [38, 39]. LCGM is highly flexible, allowing a variety of complexities including partially missing data, discretely scaled repeated measures or time-varying covariates. It also has been accepted as an appropriate methodology for better understanding how LS changes longitudinally progress over time. In growth models, at least three measurement time points are required for proper estimation, and four or five measurement time points are preferable in order to estimate more complex models involving trajectories following cubic or quadratic trends [38]. Since our data contained five measurement time points, it is sufficient to obtain robust estimation in our trajectory analysis.

The LCGM model assumes that all individuals within a class follow a pattern of change over time homogeneously. i.e., each distinct subgroup of individuals follow a pattern of LS change over time that has a unique longitudinal developmental trajectory. *PROC TRAJ,* a SAS software process, was used to fit LCGM. The procedure employs an imputation technique to assign values for missing data. As goodness of fit criteria, the Bayesian Information Criterion (BIC) and Akaike Information Criterion (AIC) were obtained for each model and compared to select the best model. Labeling subjects as members of a specific trajectory group was based on the maximum probability assignment rule.

After the LS trajectories were determined and individuals were assigned to a trajectory, predictors of group membership were evaluated, testing the differences in baseline characteristics between trajectory groups by Chi-square test and ANOVA analysis. Univariate and multivariable logistic regression models were then used to determine the best predictors and to quantify the strength of association between predictors and the trajectory groups. Comparisons of these characteristics were also made between the reference trajectory groups with the other trajectory groups. In the model building process, univariate logistic analyses were initially used to assess the relationship between each of the covariates and trajectory patterns; only those having significant or marginally significant association (*p*-value <0.10) with trajectory patterns were further evaluated in the multivariable logistic analyses. In the final models, containing only predictors with *p*-values < 0.05, interactions among the main predictors were also examined. Odds ratios (OR) and 95% confidence intervals (CI) were calculated. All reported *p*-values were two-tailed, and α = 0.05 was set for statistical significance. All statistical analyses were carried out using SAS software, version 9.4 (SAS Institute, Cary, NC, USA).

## Results

A total of 3517 individuals aged 65 or older met our study criteria, with subjects at follow-up assessments numbering 3047, 2652, 2326, and 1954 at 2007, 2009, 2011, and 2013, respectively (Table 1). The mean follow-up time was 5.9 years. In this study, at baseline a total of 2074 (59%) were females, and the mean age was 72 years (SD = 6 years). The majority had a spouse (61%) and had received no education or only elementary schooling (71%). In terms of household composition type, 40% were living with a spouse. Approximately 42% lived in urban areas with populations of more than 50,000, and 59% lived in a detached house. Regarding economic status, very few had private health insurance (6%), and the mean household financial adequacy index value was −151. Of the study sample, 1366 (48%) had a negative household financial adequacy index, i.e. household expenses exceed household income. Only small proportions of subjects had good physical and mental health status (18% and 29%, respectively). Demographic profile over time and the distribution of LS for each domain by repeat follow-up assessment are presented in Tables 1 and 2.

**Table 1:** Demographic characteristics of the study subjects by year

Variables	2006 (*n* = 3517)	2007 (*n* = 3047)	2009 (*n* = 2652)	2011 (*n* = 2326)	2013 (*n* = 1954)
Sex					
Male	1443 (41.0)	1251 (41.1)	1067 (40.2)	930 (40.0)	765 (39.2)
Female	2074 (59.0)	1796 (58.9)	1585 (59.8)	1396 (60.0)	1189 (60.9)
Age					
65–69	1505 (42.8)	878 (28.8)	283 (10.7)	-	-
70–74	983 (28.0)	1071 (35.2)	1170 (44.1)	941 (40.5)	466 (23.9)
75–79	587 (16.7)	641 (21.0)	704 (26.6)	792 (34.1)	857 (43.9)
≥ 80	442 (12.6)	457 (15.0)	495 (18.7)	593 (25.5)	631 (32.3)
Spouse					
Yes	2141 (60.9)	1802 (59.1)	1526 (57.5)	1296 (55.7)	1027 (52.6)
No	1376 (39.1)	1245 (40.9)	1126 (42.5)	1030 (44.3)	927 (47.4)
Education					
None	1245 (35.5)	1057 (34.7)	889 (33.5)	770 (33.2)	618 (31.6)
Grade 1–6	1252 (35.7)	1098 (36.0)	983 (37.1)	865 (37.3)	749 (38.4)
Grade 7–9	394 (11.2)	354 (11.6)	314 (11.8)	271 (11.7)	243 (12.4)
Grade 10–12	403 (11.5)	351(11.5)	306 (11.5)	261 (11.2)	213 (10.9)
Grade > 12	216 (6.15)	187 (6.1)	160 (6.1)	155 (6.8)	130 (6.6)
Residential area					
Urban	1490 (42.4)	1256 (41.2)	1058 (39.9)	908 (39.0)	748 (38.3)
Rural	2027 (57.6)	1791 (58.8)	1594 (60.1)	1418 (61.0)	1206 (61.7)
Housing type					
Detached House	2068 (58.8)	1768 (58.0)	1526 (57.5)	1394 (59.9)	1172 (60.0)
Apartment	962 (27.4)	849 (27.9)	762 (28.7)	674 (29.0)	568 (29.1)
Others	487 (13.9)	430 (14.1)	364 (13.7)	258 (11.1)	214 (11.0)
Household composition					
Single adult	628 (17.9)	632 (20.8)	645 (24.3)	600 (25.8)	601 (30.8)
Couple	1402 (39.9)	1246 (41.1)	1079 (40.7)	923 (39.7)	807 (41.3)
Others	1483 (42.2)	1156 (38.1)	928 (35.0)	802 (34.1)	546 (27.9)
Physical health					
Poor	2212 (62.9)	1974 (64.8)	1655 (62.5)	1184 (50.9)	1043 (53.4)
Fair	662 (18.8)	570 (18.7)	545 (20.6)	785 (33.8)	591 (30.3)
Good	643 (18.3)	503 (16.5)	448 (16.9)	356 (15.3)	320 (16.4)
Mental health					
Poor	1352 (38.5)	1182 (38.8)	723 (27.3)	582 (25.0)	507 (26.0)
Fair	1149 (32.7)	1043 (34.2)	881 (33.2)	944 (40.6)	821 (42.0)
Good	1015 (28.9)	822 (27.0)	1047 (39.5)	799 (34.4)	626 (32.0)
Private health insurance					
Yes	215 (6.1)	228 (7.5)	160 (6.0)	80 (3.4)	65 (3.3)
No	3302 (93.9)	2819 (92.5)	2492 (94.0)	2245 (96.6)	1889 (96.7)
Financial stress index					
≥ 0%	1461 (51.7)	1314 (49.5)	1100 (52.0)	1121 (54.9)	952 (52.3)
− 100% - 0	1054 (37.3)	1055 (39.8)	750 (35.4)	572 (28.0)	699 (38.4)
< −100%	312 (11.0)	285 (10.7)	266 (12.6)	350 (17.1)	170 (9.3)

(n;%)

**Table 2:** Life satisfaction score by domain and year

Life Satisfaction^a^	2005	2007	2009	2011	2013
Health					
1	781 (22.2)	535 ((17.6)	461 (17.4)	207 (8.93)	130 (6.6)
2	1455 (41.4)	1365 (44.8)	935 (35.3)	693 (29.8)	614 (31.4)
3	662 (18.8)	658 (21.6)	821 (31.0)	930 (40.0)	844 (43.2)
4	582 (16.6)	470 (15.4)	413 (15.6)	475 (20.4)	352 (18.0)
5	35 (1.0)	19 (0.6)	19 (0.7)	21 (0.9)	14 (0.7)
Finance					
1	449 (12.8)	291 (9.6)	299 (11.3)	164 (7.1)	115 (5.9)
2	1335 (38.0)	1145 (37.6)	949 (35.8)	718 (30.9)	530 (27.1)
3	1153 (32.8)	1072 (35.2)	957 (36.1)	962 (41.4)	904 (46.3)
4	552 (15.7)	520 (17.1)	429 (16.2)	449 (19.3)	372 (19.0)
5	26 (0.7)	19 (0.6)	17 (0.6)	33 (1.4)	33 (1.7)
Housing					
1	95 (2.7)	54 (1.8)	54 (2.0)	31 (1.3)	20 (1.0)
2	614 (17.5)	376 (12.3)	259 (9.8)	287 (12.3)	161 (8.2)
3	1086 (30.9)	974 (32.0)	1010 (38.1)	1011 (43.5)	902 (46.2)
4	1595 (45.4)	1544 (50.7)	1263 (47.6)	926 (39.8)	816 (41.8)
5	127 (3.6)	99 (3.3)	66 (2.5)	71 (3.1)	55 (2.8)
Family Relationships					
1	47 (1.3)	19 (0.7)	24 (0.9)	8 (0.4)	13 (0.7)
2	218 (6.2)	135 (4.6)	97 (3.8)	130 (5.8)	86 (4.5)
3	980 (27.9)	897 (30.5)	900 (34.8)	971 (43.1)	739 (38.6)
4	2073 (59.1)	1735 (59.0)	1476 (57.0)	1075 (47.7)	950 (49.6)
5	190 (5.4)	157 (5.3)	92 (3.6)	70 (3.1)	126 (6.6)
Neighbor Relationships					
1	20 (0.6)	11 (0.4)	35 (1.3)	17 (0.7)	6 (0.3)
2	216 (6.1)	159 (5.2)	124 (4.7)	146 (6.3)	117 96.0)
3	955 (27.2)	1001 (32.9)	1138 (43.2)	1105 (47.9)	928 (47.7)
4	2087 (59.4)	1721 (56.5)	1283 (48.7)	986 (42.7)	797 (40.9)
5	238 (6.8)	155 (5.1)	56 (2.1)	53 (2.3)	99 (5.1)

^**a**^1 = very unsatisfactory, 2 = unsatisfactory, 3 = fair, 4 = satisfactory, 5 = very satisfactory

**Fig. 1:**
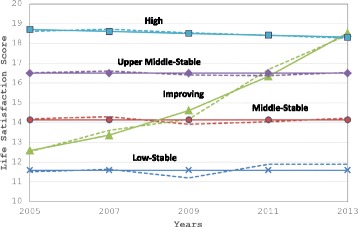
Life satisfaction Trajectories. The solid line indicates the observed mean LS score; the dashed line indicates the predicted LS score

In this study, five life satisfaction trajectory groups (TG) were identified across the eight years of follow-up by latent class growth modeling: “low-stable (TG1)”, “middle-stable (TG2)”, “improving (TG3)”, “upper middle-stable (TG4)”, and “high (TG5)”. Three trajectories were flat, one trajectory was linear, and one trajectory was quadratic (Fig. 1).

The first trajectory, TG1 (*n* = 282; 8%), was low-stable, showing low levels of LS at all time points, with an average LS score of 11.5. The second trajectory, TG2 (*n* = 1146; 32.6%), was middle-stable, which revealed steady middle LS scores over time with an average LS value of 13.9. The third trajectory, TG3 (*n* = 75; 2.1%), started with the second lowest LS but improved over time to higher LS scores at the end, changing from 12 to 19 (Table 3). The fourth trajectory, TG4 (*n* = 1653; 47%), was upper middle-stable, indicating steady upper middle-stable LS scores over time, with an average LS score of 16.6. The fifth trajectory, TG5 (*n* = 361; 10.3%), was high, showing high LS scores at the beginning and only a slight decline over time, the average LS score declining from 19.2 to 18.5 (Fig. 1). Overall, the upper middle-stable LS trajectory group had the highest percentage of participants (47%), while the improving LS trajectory group had the lowest percentage of participants (*n* = 75; 2.1%).

**Table 3:** Distribution of Baseline Characteristics by Life Satisfaction Trajectory Groups

Variables	Low-stable (n = 282)	Middle-stable (*n* = 1146)	Improving (*n* = 75)	Upper middle-stable (*n*= 1653)	High-stable (*n* = 361)	*p*-value^a^
Sex						<0.0001
Male	104 (36.9	391 (34.1)	32 (42.7)	717 (43.4)	199 (55.1)	
Female	178 (63.1)	755 (65.9)	43 (57.3)	936 (56.6)	162 (44.9)	
Age						<0.0001
65–69	92 (32.6)	446 (38.9)	46 (61.3)	738 (44.7)	183 (50.7)	
70–74	81 (28.7)	322 (28.1)	16 (21.3)	458 (27.7)	106 (29.4)	
75–79	65 (23.1)	209 (18.2)	9 (12.0)	267 (16.2)	37 (10.3)	
≥ 80	44 (15.6)	169 (14.8)	4 (5.3)	190 (11.5)	35 (9.7)	
Spouse						<0.0001
Yes	135 (47.9)	609 (53.1)	55 (73.3)	1068 (64.6)	274 (75.9)	
No	147 (52.1)	537 (46.9)	20 (26.7)	585 (35.4)	87 (24.1)	
Education						<0.0001
None	127 (45.2)	962 (43.4)	21 (28.0)	537 (32.5)	64 (17.8)	
Grade 1–6	97 (34.5)	398 (34.8)	38 (50.7)	614 (37.2)	105 (29.2)	
Grade 7–9	28 (10.0)	104 (9.1)	10 (13.3)	201 (12.2)	51 (14.2)	
Grade 10–12	24 (8.5)	103 (9.0)	5 (6.7)	203 (12.3)	68 (18.9)	
Grade > 12	5 (1.8)	42 (3.7)	1 (1.3)	96 (5.8)	72 (20.0)	
Residential area						0.0003
Urban	148 (52.5)	503 (43.9)	22 (29.3)	666 (40.3)	151 (41.8)	
Rural	134 (47.5)	643 (56.1)	53 (70.7)	987 (59.7)	210 (58.2)	
Housing type						<0.0001
Detached House	184 (65.3)	668 (58.3)	49 (65.3)	972 (58.8)	195 (54.0)	
Apartment	39 (13.8)	268 (23.4)	22 (29.3)	493 (29.8)	140 (37.8)	
Others	59 (20.1)	210 (18.3)	4 (5.3)	188 (11.4)	26 (7.2)	
Household composition						<0.0001
Single adult	90 (31.9)	252 (22.1)	12 (16.2)	242 (14.6)	32 (8.9)	
Couple	83 (29.4)	372 (32.6)	31 (41.9)	719 (43.5)	197 (54.6)	
Others	109 (39.7)	519 (45.4)	31 (41.9)	692 (41.9)	132 (36.6)	
Physical health						<0.0001
Poor	256 (90.8)	926 (80.8)	63 (84.0)	895 (54.1)	72 (19.9)	
Fair	17 (6.0)	143 (12.5)	9 (12.0)	399 (24.1)	94 (26.0)	
Good	9 (3.2)	77 (6.7)	3 (4.0)	359 (21.7)	195 (54.0)	
Mental health						<0.0001
Poor	201 (71.3)	613 (53.5)	46 (61.3)	461 (27.9)	31 (8.6)	
Fair	65 (23.1)	369 (32.2)	24 (32.0)	616 (37.3)	75 (20.8)	
Good	16 (5.7)	163 (14.2)	5 (6.7)	576 (34.9)	255 (70.6)	
Private health insurance						<0.0001
Yes	5 (1.8)	45 (3.9)	4 (5.3)	114 (6.9)	47 (13.0)	
No	277 (98.2)	1101 (96.1)	71 (94.7)	1539 (93.1)	314 (87.0)	
Financial stress index						<0.0001
≥ 0%	83 (37.6)	423 (46.2)	31 (49.2)	732 (54.8)	192 (66.0)	
− 100% - 0	91 (41.2)	374 (40.8)	27 (42.9)	476 (35.6)	86 (29.6)	
< −100%	47 (21.3)	119 (13.0)	5 (7.9)	128 (9.6)	13 (4.5)	
Life satisfaction score						<0.0001@
2005	11.0 (±2.3)	13.8 (±1.9)	11.9 (±2.2)	16.6 (±1.9)	19.2 (±1.9)	
2007	11.2 (±1.9)	14.1 (±1.7)	13.4 (±2.0)	16.7 (±1.8)	19.2 (±1.6)	
2009	10.9 (±2.1)	13.7 (±1.9)	13.9 (±2.2)	16.4 (±1.8)	19.0 (±1.8)	
2011	11.6 (±2.1)	13.9 (±2.2)	17.4 (±2.4)	16.4 (±2.1)	18.8 (±2.2)	
2013	11.6 (±2.0)	14.0 (±2.1)	19.0 (±1.9)	16.6 (±2.1)	18.5 (±2.1)	

^a^Global test for differences among the five trajectory groups by Chi-square test; ^@^ ANOVA analysis.

(n, %; mean, ±s.d)

(n;%)

The high LS trajectory group (TG5) had a high percentage of participants who were 65 to 69 years old at baseline. Subjects in this group also tended to have high education levels, to live with a spouse, to be in good health, and to be financially secure. Descriptive characterization and mean LS scores across the five trajectory groups are presented in the Table 3. In examining characteristics of trajectory groups, global tests from the Chi-square test and ANOVA procedures showed that all selected variables were significantly different among the five groups (Table 3).

Several sets of logistic regression models were generated to compare subject characteristics between the respective trajectories, varying the reference group. In the first set of models, the high LS group, TG5, was used as the reference trajectory; the odds ratios with 95% CI are shown in Tables 4 and 5. Univariate logistic regression showed that male sex, high education, financial adequacy, living with a spouse, living in a rural area, not living in a detached house, and good physical and mental health were all significant predictors for high LS trajectory (Table 4). However, multivariable analysis indicated that individuals in the high LS trajectory group were more likely to have higher education, good physical health, and good mental health than those in all other trajectory groups (Table 5). Sex, age, having a spouse, having private health insurance, and residential area were not significant predictors for the high LS trajectory group in the adjusted model. Financial adequacy was a significant predictor for high LS trajectory, compared with all other groups except the improving LS group (Table 5).

**Table 4:** Univariate Logistic Regression Analyses. Estimation of odds ratio (OR) and 95% confidence interval (C.I.). High-stable life satisfaction as reference trajectory group

Variables	Low-stable (*n* = 282) OR (95% CI)	Middle-stable (*n* = 1146) OR (95% CI)	Improving (*n* = 75) OR (95% CI)	Upper middle-stable (*n* = 1653) OR (95% CI)
Sex				
Female vs Male	2.10 (1.53–2.89)	2.37 (1.87–3.02)	1.65 (0.99–2.73)	1.60 (1.28–2.02)
Age				
70–74 vs 65–69	1.52 (1.04–2.23)	1.25 (0.94–1.65)	0.60 (0.32–1.18)	1.07 (0.82–1.40)
75–79 vs 65–69	3.49 (2.17–5.62)	2.32 (1.57–3.42)	0.97 (0.44–2.15)	1.79 (1.22–2.62)
≥80 vs 65–69	2.50 (1.50–4.16)	1.98 (1.33–2.96)	0.46 (0.15–1.34)	1.35 (0.91–2.00)
Spouse				
No vs Yes	3.43 (2.45–4.80)	2.78 (2.13–3.63)	1.10 (0.65–2.02)	1.73 (1.33–2.24)
Education				
Grade 1–6 vs None	0.47 (0.31–0.70)	0.49 (0.35–0.69)	1.02 (0.60–2.04)	0.70 (0.50–0.97)
Grade > 6 vs None	0.15 (0.10–0.23)	0.17 (0.12–0.23)	0.26 (0.23–0.52)	0.31 (0.23–0.43)
Residential area				
Rural vs Urban	0.65 (0.48–0.89)	0.92 (0.72–1.17)	1.73 (1.01–2.97)	1.07 (0.85–1.34)
Housing type				
Apartment vs Detached	0.15 (0.09–0.24)	0.24 (0.16–0.36)	0.42 (0.20–0.90)	0.48 (0.32–0.72)
Others vs Detached	0.29 (0.18–0.47)	0.50 (0.33–0.76)	0.63 (0.29–1.35)	0.69 (0.46–1.05)
Household composition				
Couple vs Single	0.30 (0.20–0.44)	0.56 (0.43–0.72)	0.63 (0.36–1.08)	0.71 (0.55–0.90)
Others vs Single	2.41 (1.45–3.98)	2.36 (1.52–3.65)	0.61 (0.20–1.84)	1.45 (0.94–2.25)
Physical health				
Fair vs Poor	0.05 (0.03–0.09)	0.12 (0.08–0.17)	0.11 (0.05–0.24)	0.34 (0.25–0.47)
Good vs Poor	0.01 (0.01–0.03)	0.03 (0.02–0.04)	0.02 (0.01–0.06)	0.15 (0.11–0.20)
Mental health				
Fair vs Poor	0.13 (0.08–0.22)	0.25 (0.16–0.39)	0.21 (0.11–0.41)	0.55 (0.36–0.85)
Good vs Poor	0.01 (0.01–0.02)	0.03 (0.02–0.05)	0.01 (0.01–0.04)	0.15 (0.10–0.23)
Private health insurance				
Yes vs No	0.12 (0.05–0.31)	0.27 (0.18–0.42)	0.38 (0.13–1.08)	0.49 (0.35–0.71)
Financial stress index				
(−100% - 0) vs (≥0%)	2.45 (1.66–3.62)	1.97 (1.48–2.64)	1.94 (1.09–3.46)	1.45 (1.10–1.92)
(< −100%) vs (≥0%)	8.36 (4.30–16.30)	4.15 (2.29–7.55)	2.38 (0.79–7.15)	2.58 (1.43–4.67)

**Table 5:** Multivariable Logistic Regression Analyses. Estimation of odds ratio (OR) and 95% confidence interval (C.I). High-stable life satisfaction as reference trajectory group

Variables	Low-stable (*n* = 282) OR (95% CI)	Middle-stable (*n* = 1146) OR (95% CI)	Improving (*n* = 75) OR (95% CI)	Upper middle-stable (*n* = 1653) OR (95% CI)
Sex				
Female vs Male	-	-	-	-
Age				
70–74 vs 65–69	-	-	-	-
75–79 vs 65–69				
≥ 80 vs 65–69				
Spouse				
No vs Yes	-	-	-	-
Education				
Grade 1–6 vs None	1.08 (0.40–2.41)	0.80 (0.49–1.31)	0.95 (0.40–2.24)	0.72 (0.49–1.08)
Grade > 6 vs None	0.31(0.14–0.72)	0.31 (0.19–0.51)	0.32 (0.13–0.80)	0.41 (0.28–0.59)
Residential area				
Rural vs Urban	-	-	-	-
Housing type				
Apartment vs Detached	0.44 (0.20–0.95)	0.84 (0.54–1.29)	-	1.07 (0.79–1.44)
Others vs Detached	1.60 (0.56–4.57)	2.93 (1.51–5.70)		1.99 (1.19–3.34)
Household composition				
Couple vs Single	0.23 (0.10–0.57)	0.25 (0.14–0.44)	-	-
Others vs Single	0.56 (0.22–1.45)	0.49 (0.27–0.88)		
Physical health				
Fair vs Poor	0.07 (0.03–0.18)	0.11 (0.07–0.18)	0.12 (0.05–0.31)	0.33 (0.22–0.49)
Good vs Poor	0.08 (0.03–0.22)	0.09 (0.06–0.15)	0.08 (0.02–0.31)	0.28 (0.19–0.40)
Mental health				
Fair vs Poor	0.40 (0.17–0.96)	0.69 (0.38–1.24)	0.53 (0.24–1.20)	0.97 (0.58–1.62)
Good vs Poor	0.04 (0.02–0.10)	0.11 (0.06–0.19)	0.04 (0.01–0.12)	0.35 (0.21–0.56)
Private health insurance				
Yes vs No	-	-	-	-
Financial stress index				
(−100% - 0) vs (≥0%)	1.72 (0.88–3.34)	1.37 (0.91–2.08)	-	1.31 (0.97–1.78)
(< −100%) vs (≥0%)	5.52 (1.76–17.30)	3.79 (1.68–8.52)		2.57 (1.38–4.80)

The individuals in the low-stable trajectory group, the worst outcomes, were specifically reviewed. Not only were in these individuals in poorer physical and mental health, but they were also more likely to be living alone and be more financially stressed than those in the upper middle-stable (Table 6) or high trajectory groups (Table 5). The individuals in the low-stable group were older than those in the upper middle-stable or improving group (Table 6). Compared with the upper middle-stable group, the low-stable group was less likely to live in a rural area than an urban area (OR = 0.47, 95% CI: 0.33–0.66).

**Table 6:** Multivariable Logistic Regression Analyses. Estimation of odds ratio (OR) and 95% confidence interval (C.I)

Variables	Low-stable^a^ (*n*= 282) OR (95% CI)	Middle-stable^a^ (*n*= 1146) OR (95% CI)	Improving^a^ (*n*= 75) OR (95% CI)	Low-stable^b^ (*n*= 282) OR (95% CI)
Sex				
Female vs Male	-	-	-	-
Age				
70–74 vs 65–69	1.45 (0.97–2.17)	-	0.57 (0.32–1.03)	2.67 (1.34–5.35)
75–79 vs 65–69	1.94 (1.25–3.02)	-	0.49 (0.24–1.03)	3.07 (1.34–7.03)
≥80 vs 65–69	1.47 (0.87–2.47)	-	0.31 (0.11–0.87)	4.88 (1.57–15.2)
Spouse				
No vs Yes	-	-	-	2.27 (1.24–4.15)
Education				
Grade 1–6 vs None	-	-	-	-
Grade > 6 vs None				
Residential area				
Rural vs Urban	0.47 (0.33–0.66)	0.75 (0.62–0.91)	-	0.35 (0.19–0.64)
Housing type				
Apartment vs Detached	0. 47 (0.30–0.75)	0.84 (0.68–1.06)	-	0.40 (0.20–0.79)
Others vs Detached	1.50 (0.95–2.38)	1.63 (1.24–2.15)		2.52 (0.84–7.60)
Household composition				
Couple vs Single	0.41 (0.27–0.62)	0.51 (0.39–0.65)	-	-
Others vs Single	0.52 (0.34–0.79)	0.72 (0.56–0.93)		
Physical health				
Fair vs Poor	0.21 (0.11–0.40)	0.42 (0.32–0.55)	-	-
Good vs Poor	0.28 (0.13–0.62)	0.36 (0.26–0.51)		
Mental health				
Fair vs Poor	0.32 (0.22–0.47)	0.59 (0.47–0.73)	0.38 (0.23–0.63)	-
Good vs Poor	0.12 (0.06–0.21)	0.30 (0.23–0.40)	0.08 (0.03–0.22)	
Private health insurance				
Yes vs No	-	-	-	-
Financial stress index				
(−100% - 0) vs (≥0%)	1.45 (1.01–2.08)	1.26 (1.03–1.53)	-	-
(< −100%) vs (≥0%)	3.11 (1.94–4.98)	1.55 (1.15–2.09)		

^a^Upper middle-stable life satisfaction as reference trajectory group

^b^Improving life satisfaction as reference trajectory group

As the upper-middle stable trajectory goup was the largest, subjects in the low-scoring, less populated trajectories were compared to this group, aiming to identify characteristics that predict a less satisfied, less typical trajectory. The upper middle-stable group was noted to be younger than the low stable group and less likely to live in a rural area than an urban one (OR = 0.47, 95% CI: 0.33–0.66). The middle-stable trajectory group was also less likely to live in a rural area than an urban one (OR = 0.75, 95% CI: 0.62, 0.91), although no age difference was observed with this comparison.

Finally, a small group of individuals assigned to a unique trajectory, having scores that were similar to the low stable group at baseline, but rising to levels approximating those of the high stable group at the end of the study, were examined. Compared to subjects with consistently low scores, those in the improving trajectory were younger, more likely to have a spouse, and more likely to live in a rural area. Neither physical nor mental health was a significant predictor in differentiating between the low-stable and the improving trajectory groups.

## Discussion

Using longitudinal data from the KReIS, we were able to investigate groups of older people in Korea, showing distinct trajectories of life satisfaction, and determine characteristics predicting specific trajectory group membership. To identify these heterogeneous groups, we analyzed trajectories of life satisfaction with latent class growth modelling. The primary hypothesis in our study, that LS in the older adults follows distinct trajectories (a slightly declined group, an increased group, and three stable groups), was confirmed. Our study showed five trajectories: low-stable LS trajectory, middle-stable LS trajectory, improving LS trajectory, upper middle-stable LS trajectory, and high LS trajectory. The second hypothesis, that demographic, physical, and environmental characteristics are predictors of life satisfaction trajectory membership, was also partially confirmed.

Life satisfaction in the older adults is not uniform, but rather follows distinct trajectories [7–9, 12, 40]. For the most part, the LS trajectories identified in our study are similar to the trajectories found in Taiwanese older adults. Hsu and Jones [40] applied group-based trajectory analysis and identified four groups: successful aging, usual aging, declining health, and care demanding. In their study, the heterogeneity of aging trajectories reflected differences in life satisfaction. Both Hsu’ study and our study found that members of high LS trajectory (the successful aging group in Hsu’s study) had few physical and mental health issues, stable social support, and high economic satisfaction. The upper middle-stable trajectory group in our study is similar to the usual aging group in Hsu’s study and made up the largest percentage of respondents. Although there were similarities in characteristics between Hsu’s trajectory group and our trajectory group, Hsu could not identify a more dynamic improving life satisfaction group similar to TG3. In our study, this dynamic group (TG3) only comprised a small proportion of subjects (2.2%). Our study, as well as Hsu’s study did not show a sharp decline in LS among older adults, which differs from German and British studies [7, 9]. This discrepancy might be due to cultural and social differences between Western and Asian countries. LS and happiness as subjective well-being will differ from one culture to the others to some degree. For example, living with children is very common in Asian culture, and it is associated with good self-perceived health and low prevalence of depressive symptoms in a culture where family interdependence is highly valued.

The literature shows that physical health and mental health are strongly associated with LS [10–14, 16]. Our study agrees, concluding that compared to the high LS trajectory group members, individuals who were in poor physical and mental health were more likely to be in the low-stable, middle-stable, or upper middle-stable trajectory groups. This finding was also consistent with Hsieh’s study (2003) which found that the most important life satisfaction domains were family and religion, as well as health [34]. Our finding that LS trajectory was significantly associated with physical and mental health was again consistent with the Taiwanese elderly study [15].

There are additional predictors noted in our study that have been identified by others. Our study showed that financial insecurity is related to lower life satisfaction. This result aligns with other studies which identify financial strain as an important factor for predicting LS [13, 14, 28]. Studies have also detected that spouse/partnership is an especially pertinent domain for older people’s well-being [25, 27]. In older people, having a spouse can provide important support for finances and health issues [41]. Our study showed that living with a spouse was a significant predictor in univariate analysis but was no longer significant in the multivariable analysis. Living arrangements influence health, presumably by the provision of social support and subsequent prevention of loneliness in old age.

There is, however, also discrepancy between our findings and other published research, particularly regarding two specific factors. Firstly, some studies have also found location of residence to be influential, with urban residents having higher life satisfaction than rural residents [18, 42]. It may be that older people who live in urban situations have better access to medical and health resources. However, in our study we observed that the individuals in the lower low-stable and middle-stable trajectory groups were more likely to be living in an urban area than rural area compared to those in the more satisfied upper middle-stable group. In general, living in a rural area potentially provides a relatively steady and close social network through regular contact over time, which is associated with good LS. Secondly, a few studies in the older population have also shown that females have lower LS than males [28, 43, 44]. As it would appear from the literature that females have higher LS with a higher number of social activities and a larger circle of friends, losses of these elements with advancing age are potentially felt more acutely in women; these are not significant predictors of LS among the males [21, 41]. Our study, however, showed that sex was not a significant predictor of LS trajectory, a finding which is consistent with another recent study [6].

Trajectory study in clinical research is the longitudinal investigation of outcomes (for example, health, disease, depression, pain, life satisfaction, health care utilization, etc.) of subjects over time. Subjects in a trajectory study could be persons, families, groups, communities, or populations. Unlike cross-sectional studies, trajectory research focuses on change over time in one subject and thus is more dynamic. Our study identified five distinct LS trajectory groups among the elderly using the latent growth modeling approach and investigated association between LS trajectory groups and risk factors. These findings support the association between risk factors such as education, mental/physical health, financial independence, living environments, and life satisfaction of the elderly. We found that one the most influential factors for high life satisfaction in older adults is the maintainance of physical and mental health. The study also demonstrated that economic security and living environment are important for LS of the elderly.

The main limitation of our study is that the selected variables in the study obviously do not cover all potential health and psychosocial aspects that may be of relevance to life satisfaction. Our data does not contain specific health-related variables and medically-based health measures, including chronic disease, anxiety, depression, etc., which would have further improved our understanding of life satisfaction in older adults. Another limitation is related to the selection of potential predictors of life satisfaction. As indicated in many studies, religion, leisure activity, and measurements of social support and family support are additional important factors associated with life satisfaction in older adults. Unfortunately, such information was not available in our study data and, thus, the relative importance of certain predictors for life satisfaction remains unobserved. Additionally, self-report questionnaires were used when collecting the data analyzed in our study, another validity limitation.

However, our study has the following strengths. Firstly, whereas many previous studies collected cross-sectional data from a non-representative sample population, our study used longitudinal data with a large number of participants from a nationally representative sample of Korean adults. In addition, whereas most cross-sectional studies have focused on overall mean changes across all participants, we were able to identify different trajectories and to determine variables that predict membership in these trajectories. Secondly, we were able to assess life satisfaction trajectories over the eight year study time with sufficient measurement time points. With our long follow-up data and appropriate trajectory analysis methodology, we were able to define the distinctive LS groups by their trajectories over time. Thirdly, most studies on LS have only analyzed a single domain; however, LS is correlated with multidimensional successful aging [45, 46]. Studies suggest that various life domains differentially contribute to overall life satisfaction [2, 47–49]. In our study, the LS outcome measurement covered five domains including health, finance, housing, neighbor relationships, and family relationships. Thus, our study results are potentially more comprehensive than other studies.

Our study contributes to the literature on the associations between physical and mental health status, living environment, housing, family support and life satisfaction in older adults. Physical, mental, social, and financial aspects potentially interact with each other to determine LS in the older population. These factors should be considered when addressing the support or care of elderly people with an aim - to improve their life satisfaction. Generally, increasing rates of a variety of chronic diseases can be expected to translate into frequently observed declines in physical and mental function, social activity, and networking. These declines, in turn, will affect levels of life satisfaction in elderly individuals. The implication of our findings can be adopted by the government agencies, policy decision makers, and health professionals to: (i) identify the low-stable LS group among the old adults population, individuals who are most likely to be old-old (aged 80 above), living alone, in poor physical or mental health, and financially vulnerable; and (ii) design welfare programs, community resources, policies, and regulations for successful aging with respect to LS.

The associations between specific factors and low LS trajectories provide direction for the improvement of LS support efforts. Family members, health-care providers, and government agency must recognize the varying need for support based on types of living environment (housing, living arrangement, activities). Considering that low LS is related to poor physical and mental health, living alone (feeling lonely), and poor financial resources, social work interventions in terms of health education directed towards physical, psychological, social, and economic aspects of wellbeing are necessary, especially for those at risk of low LS.

Government agencies must re-evaluate pension policies affecting the low LS population, and public interventions to increase family-based and community-based support for elderly must be implemented. Health care professionals should also focus on ways of promoting physical and social activities through various community programs for older adults.

## Conclusion

Our study found that age related changes in LS in the older population are not uniform, but heterogeneous. Five trajectories of life satisfaction in the elderly Korean population were identified based on latent growth mixture modelling analysis. Our study showed that having higher education, having good physical and mental health, not living alone, and being financially secure predict higher LS. Our study provides a more complete understanding of LS trajectory in the older adults and more clearly identifies the subgroups of individuals at risk for lower LS. Globally, an ongoing increase in the proportion of older adults within the population will require considerable medical, social, and economic resources in the future. LS trajectory results have policy implications, allowing identification of individuals most likely to be in need of disease/care management programs and resource allocation. By identifying the low-stable LS group, community needs-based interventions can be implemented to improve physical and mental health. The promotion and stimulation of health and activity via the multifaceted influences identified in this study is anticipated to help make life worth living in this most vulnerable group.

## Abbreviations

KreIS: Korean Retirement and Income Study
LCGM: Latent Class Growth Modelling
LS: Life Satisfaction
